# Antiinflammatory, Diuretic and Antimicrobial Activities of *Rungia pectinata* Linn. and *Rungia repens* Nees

**DOI:** 10.4103/0250-474X.45418

**Published:** 2008

**Authors:** S. R. Swain, B. N. Sinha, P. N. Murthy

**Affiliations:** * Institute of Pharmacy and Technology, Salipur, Cuttack-754 202, India; 1 Birla Institute of Technology, Mesra, Ranchi-835 215, India; 2 Royal College of Pharmacy and Health Sciences, Berhampur-760 001, India

**Keywords:** Antiinflammatory, diuretic, antimicrobial, *Rungia pectinata* and *Rungia repens*

## Abstract

The hydroalcoholic extracts prepared from leaves of *Rungia pectinata* and *Rungia repens* were investigated for antiinflammatory and diuretic activity in wistar rats. The results obtained were compared with that of standard drug aspirin and frusemide for their antiinflammatory and diuretic activity respectively. The acute toxicity study was also carried out using adult swiss albino mice of either sex which indicates the safety of the extracts even at a dose of 4000 mg/kg. *R. pectinata* showed better anti-inflammatory activity than *R. repens*. In the present study, it was demonstrated that hydroalcoholic extracts of both *R. repens* and *R. pectinata* produce diuretic effect by increasing the excretion of Na^+^, K^+^ and Cl^−^. Results showed that *R. repens* is most effective in increasing urinary electrolyte concentration of Na^+^ and K^+^ ions. The antimicrobial potency of the aerial parts of *Rungia pectinata* and *Rungia repens* have been studied using the petroleum ether, benzene, chloroform, acetone and ethanol extract against a wide number of bacteria and fungi by disc diffusion method. The ethanol extract at a concentration of 30 to 60 μg/disc showed significant activity against the bacteria and fungus investigated. All the extracts of *R. pectinata* and *R. repens* have got moderate action but chloroform and acetone extracts of *R. repens* and ethanol extract of *R. pectinata* have got significant activity against *Trichophyton mentagrophytes*.

*Rungia pectinata* (*Acanthaceae*) is a much branched, procumbent or erect herb found as a weed throughout the warmer parts of India^1^,^2^. The juice of the leaves is considered cooling and aperient, and is given to children suffering from smallpox. Bruised leaves are applied to contusions to relieve pain and reduce swelling. In Bihar, the roots are used as a febrifuge by the tribal population^3^,^4^. There are also reports that it is of use as diuretic and vermifuge^5^.

*Rungia repens* (*Acanthaceae*), a spreading decumbent herb found through out India mostly as a weed in moist places^1^,^2^. The herb is dried and pulverized for use in the treatment of cough and fever; it is also credited with vermifugal and diuretic properties^3^. Fresh, bruised leaves are mixed with castor oil and applied to scalp to cure *Tinea capitis*, a scaly fungoid infection, usually occurring amongst children^4^–^7^. Investigation on the flavonoid pigments in ivory-white and pale yellow flowers showed the presence of luteolin and chrysoeriol (3'-o-methylluteolin) and their glucosides^8^. Flowers with deep yellow tubular portion and bluish pink spots contain isosalipurposide (2'-glucosyloxy-4,4',6'-trihydroxychalcone, mp 152-53°), occurring with luteolin and its 7-glucoside; the bluish pink colour is due to the presence of delphinidin-3,5-diglucoside^9^.

The leave of *Rungia pectinata* and *Rungia repens* were obtained from nearby areas of Salipur, Orissa and identified at Botanical Survey of India, Howrah. Their voucher specimens were deposited in the herbarium. All other chemicals and reagents used were of analytical grade. The experiment protocols were approved by the Institutional Animal Ethics Committee prior to the conduct of the animal experiments (1053/ac/07/CPCSEA).

Air-dried, powdered plant materials were Soxhlet extracted separately for 75 h in a mixture of ethanol and water (50:50). The hydroalcoholic extracts were concentrated and dried separately using a rotary flash evaporator to give solid residue. The yields were 8.52 and 7.64% w/w for *R. repens* and *R. pectinata* respectively. For antimicrobial activity air-dried, powdered plant material was Soxhlet extracted in petroleum ether, benzene, chloroform, acetone and ethanol in increasing order of polarity. The different extracts were concentrated and dried using a rotary flash evaporator to give solid residues. The yield was 2.21, 2.08, 4.90, 4.03, 4.18 and 2.74, 3.01, 2.65, 3.25, 4.12% w/w for *R. repens* and *R. pectinata,* respectively with petroleum ether, benzene, chloroform, acetone and ethanol. Phytochemical screening gave positive test of phytosterols, terpenes, tannins, flavonoids and carbohydrates^10^.

The acute toxicity of the extracts was determined by the method of Lorke using the oral route^11^. Adult Swiss albino mice of either sex, weighing between 20 to 25 g were divided into 9 groups of six animals each. The control group received 2 ml/kg distilled water orally. The other groups received the extracts at dose levels of 100, 200, 400, 800, 1000, 2000, 3000 and 4000 mg/kg in distilled water as suspension through oral route. After administration of dose the animals were observed continuously for first 4 h for behavioral changes and for mortality, if any, at the end of 24, 48 and 72 h, respectively.

Wistar rats (140-190 g) of both sexes were used for inducing edema in their paw was studied by the method of Winter *et al*^12^. The animals were housed in cages under standard laboratory condition. They had free access to standard diet and water. The animal were divided into groups of six animals each and fasted for 12 h before the experiment.

The initial right hind paw volume of the rats were measured using a plethysmometer (Ugo Basile) and then 0.1 ml of 1% (w/v) carrageenan was subcutaneously injected into the subplantar region of the right hind paw. The volume of right hind paw was measured at 1, 2, 3, 4, and 5 h after carrageenan injection, and the edema volume was determined. The data were expressed as paw volume (ml), compared with the initial hind paw volume of each rat. Co solvent, hydroalcoholic extracts (200, 400, 800 mg/kg) of *R. pectinata, R. repens* as suspension in distilled water and aspirin (200 mg/kg) was orally administered 30 min before carrageenan injection. Each group comprised of 6 rats. The group received co solvent was treated as control.

The diuretic activity in rats was studied by the method as described by Lipschitz *et al*^13^. Male Wistar rats weighing 100-200 g were used. Three animals per group were placed in metabolic cages provided with a wire mesh bottom and a funnel to collect the urine. Stainless-steel sieves were placed in the funnel to retain feces and to allow the urine to pass. The rats were fed with standard diet (pellets) and water *ad libitum*. Fifteen hours prior to the experiment food and water were withdrawn. Three animals were placed in one metabolic cage. For screening procedures two groups of three animals were used for one dose of the test compound. The test compound (hydroalcoholic extract of *R. pectinata* and *R. repens*) was given orally at a dose of 400 mg/kg and 800 mg/kg in 5.0 ml water/kg body weight. Two groups of three animals each received orally frusemide (20 mg/kg) and served as positive control. Additionally, 5 ml of 0.9% NaCl solution per 100 g body weight was given by gavage. The urine volume during 5 h and 24 h was measured and urine electrolyte estimation was carried out for Na^+^, K^+^ using flame photometer of Elico Pvt. Ltd., model CL 22D^14^ and Cl^−^ was estimated by titration^15^–^17^. Urine volume excreted per 100 g body weight was calculated.

All results are expressed as mean±standard error. The data was analyzed statistically using ANOVA followed by Dunnett's Multiple Comparison Test using SPSS 10.0 statistical software. The level of significance was fixed at 5%.

The *In vitro* antimicrobial activity of different extracts of the *R. pectinata* and *R. repens* were studied by disc diffusion method^18^,^19^. All the extracts at the concentration of 30 μg/ml and 60 μg/ml in dimethylformamide were tested against the bacteria *Escherichia coli, Staphylococcus pyogenes, Staphylococcus aureus, Pseudomonas aeruginosa, Bacillus subtilis* and *Bacillus cerus* and fungi *Aspergillus niger, Aspergillus flavus, Candida albicans, Fusarium oxyporum* and *Fusarium solani*. The activity of the extracts was compared with the antibacterial and antifungal standards. Rifampicin and miconazole were used as antibacterial and antifungal standards, respectively. The plates were incubated at 37° for 48 h. The zone of inhibition was calculated by measuring the minimum dimention of the zone of no microbial growth around the disc. For each data an average of three independent determinations were recorded (Tables 3 and 4).

**TABLE 3:** ANTIBACTERIAL AND ANTIFUNGAL ACTIVITY OF *R. PECTINATA*

Microorganisms	Petroleum ether(μg/disc)		Benzene (μg/disc)		Chloroform (μg/disc)		Acetone (μg/disc)		Ethanol μg/disc)		Rifampicin (μg/disc)	Miconazole (μg/disc)
	
	30	60	30	60	30	60	30	60	30	60	30	10
Bacteria												
*Escherichia coli*	+	+	−	−	−	+	+	++	++	+++	+++	NT
*Staphylococcus pyogenes*	−	−	−	−	−	+	+	++	++	+++	+++	NT
*Staphylococcus aureus*	−	−	−	+	+	++	++	++	++	+++	+++	NT
*Pseudomonas aeruginosa*	−	−	+	+	−	−	+	++	+	++	+++	NT
*Bacillus subtilis*	−	−	−	−	−	+	++	++	++	++	+++	NT
*Bacillus cerus*	+	+	−	−	−	+	+	++	++	+++	++	NT
Fungi												
*Aspergillus niger*	+	++	−	+	+	++	+	++	++	+++	NT	+++
*Aspergillus flavus*	+	+	−	−	+	+	−	++	++	+++	NT	+++
*Candida albicans*	+	+	−	−	+	++	++	+++	+++	+++	NT	+++
*Fusarium oxyporum*	−	−	−	−	−	+	+	++	++	++	NT	+++
*Fusarium solani*	−	−	−	+	−	+	−	+	+	++	NT	+++
*Trichophyton mentagrophytes*	+	++	+	+	+	++	+	++	++	+++	NT	+++

Experiments were done in triplicate. Disc diameter = 4 mm. Diameter of zone of inhibition:

− =< 4;

+ = 5-10;

++ = 11-15;

+++ = > 16;

NT= not tested. DMF had not shown any antimicrobial activity against the tested organisms.

**TABLE 4:** ANTIBACTERIAL AND ANTIFUNGAL ACTIVITY OF R. REPENS

Microorganisms	Pet ether (μg/disc)		Benzene (μg/disc)		Chloroform (μg/disc)		Acetone (μg/disc		Ethanol (μg/disc)		Rifampicin (μg/disc)	Miconazole (μg/disc)
	
	30	60	30	60	30	60	30	60	30	60	30	10
Bacteria												
*Escherichia coli*	+	+	−	−	−	+	+	++	++	+++	+++	NT
*Staphylococcus pyogenes*	−	−	−	−	−	+	+	++	++	+++	+++	NT
*Staphylococcus aureus*	−	−	−	+	+	++	++	++	++	+++	+++	NT
*Pseudomonas aeruginosa*	−	−	+	+	−	−	+	++	+	++	+++	NT
*Bacillus subtilis*	−	−	−	−	−	+	++	++	++	++	+++	NT
*Bacillus cerus*	+	+	−	−	−	+	+	+	++	+++	++	NT
Fungi												
*Aspergillus niger*	+	++	−	+	+	++	+	++	++	+++	NT	+++
*Aspergillus flavus*	+	+	−	−	+	+	−	+	++	+++	NT	+++
*Candida albicans*	+	+	−	−	+	++	++	+++	+++	+++	NT	+++
*Fusarium oxyporum*	−	−	−	−	−	+	−	+	+	++	NT	+++
*Fusarium solani*	−	−	−	+	−	+	−	+	+	++	NT	+++
*Trichophyton mentagrophytes*	+	++	+	+	++	+++	+	++	++	+++	NT	+++

Experiments were done in triplicate. Disc diameter = 4mm. Diameter of zone of inhibition:

− = < 4;

+ = 5-10;

++ = 11-15;

+++ = > 16;

NT = not tested. DMF had not shown any antimicrobial activity against the tested organisms.

From the acute toxicity study it was observed that the hydroalcoholic extract of both *R. repens* and *R. pectinata* did not show any behavioral changes or mortality even at a dose of 4000 mg/kg indicative of the safety of these extracts. The antiinflammatory effect of leaf extract of *R. repens* and *R. pectinata* are shown in Table 1. *R. pectinata* showed better antiinflammatory activity than *R. repens. R. pectinata* at a dose of 200 mg/kg shown significant activity up to 3 h and further at a dose of 400 and 800 mg/kg showed significant activity up to 5 h. *R. repens* did not show any significant activity at a dose of 200 and 400 mg/kg, but showed significant activity at a dose of 800 mg up to 5 h.

**TABLE 1:** ANTIINFLAMMATORY EFFECT OF *RUNGIA PECTINATA, RUNGIA REPENS* AND ASPIRIN IN CARRAGEENAN-INDUCED RAT PAW EDEMA.

Drug	Dose (mg/kg p.o.)	Paw volume (ml)					
		
		0 h	1 h	2 h	3 h	4 h	5 h
Cosolvent		4.13±0.13	4.91±0.16	5.83±0.06	6.84±0.13	7.38±0.16	7.52±0.09
Aspirin	200	3.57±0.06	3.94±0.11*	3.96±0.08*	4.13±0.99*	4.32±0.04*	4.58±0.06*
*Rungia repens*	200	4.16±0.05	4.80±0.07	5.97±0.68	6.98±0.09	7.32±0.06	7.51±0.07
	400	3.99±0.08	4.58±0.08	5.85±0.07	6.89±0.09	7.25±0.07	7.59±0.07
	800	3.86±0.06	4.21±0.08*	5.31±0.06*	6.2±0.07*	6.61±0.08*	6.95±0.09*
*Rungia pectinata*	200	4.47±0.05	5.33±0.06*	6.56±0.07*	7.33±0.06*	7.56±0.07	7.5±0.06
	400	4.58±0.04	5.65±0.06*	6.96±0.09*	7.97±0.06*	8.27±0.04*	8.23±0.05*
	800	4.47±0.05	5.26±0.08*	6.52±0.06*	7.47±0.09*	7.54±0.08*	7.36±0.07*

Each value is mean of±SEM (n=6);

* Denotes significant difference when compared to control values at p <0.05, ANOVA followed by Dunnett's t-test.

Frusemide-treated rats showed a significant increase in volume of urine and urinary excretion of sodium, potassium and chloride (*P*<0.05) as compared to control. Higher electrolyte (sodium and potassium) excretion (*P*<0.05) was observed in *R. repens*. A significant increase in urine volume was observed at a dose of 400 mg/kg after 24 h but at a dose of 800 mg/kg the increase in urine volume was significant after 5 h as well as 24 h. *R. pectinata* at a dose of 400 mg/kg did not show significant increase in urine volume or sodium excretion but showed significant increase in electrolyte excretion (potassium and chloride) at a dose of 800 mg/kg further a significant increase in volume of urine and urinary excretion of sodium, potassium and chloride was observed. The results are summarized in Table 2.

**TABLE 2:** DIURETIC ACTIVITY OF HYDROALCOHOLIC EXTRACT OF *RUNGIA PECTINATA* AND *RUNGIA REPENS*.

Group	Treatment (n=6)	Volume of Urine (ml)		Concentration in mEq/l of Urine at 24 h		
			
		5 h	24 h	Sodium	Potassium	Chloride
I	Normal saline (25 ml/kg)	3.58±0.16	6.21±0.11	89.98±1.41	61.16±0.87	90.88±1.34
II	Frusemide (20 mg/kg)	5.4±0.21*	8.21±0.25*	120.2±1.40*	90.26±0.96*	96.33±1.11*
III	*R. repens* (400 mg/kg)	4.20±0.21	7.71±0.32*	127.25±1.02*	78.43±1.15*	91.46±0.92
IV	*R. repens* (800 mg/kg)	4.83±0.13*	7.9±0.29*	128.08±1.28*	79.58±2.81*	90.26±1.46
V	*R. pectinata* (400 mg/kg)	3.78±0.12	6.76±0.21	90.68±1.17	70.53±1.15*	97.48±1.21*
VI	*R. pectinata* (800 mg/kg)	4.33±0.19*	7.15±0.25*	126.45±1.32*	75.21±1.21*	90.20±1.44*

Each value is mean ±SEM (n = 6, that is two groups of three animals each);

* Denotes significant difference when compared to control values at P <0.05, ANOVA followed by Dunnett's t-test.

As the carrageenan-induced paw edema model was used for evaluation of antiinflammatory activity of the compounds involving several chemical mediators such as prostaglandins, serotonin, histamine and bradykinin^20^, it is possible that the active constituents in the hydroalcoholic extracts of *R. repens* and *R. pectinata* may be involved in the inhibition of some of these inflammatory mediators.

In present study, it was also demonstrated that hydroalcoholic extracts of both *R. repens* and *R. pectinata* produce diuretic effect by increasing the excretion of Na^+^, K^+^ and Cl^−^ ions. The control of plasma sodium is important in the regulation of blood volume and pressure; the control of plasma potassium is required to maintain proper function of cardiac and skeletal muscles^21^. The regulation of Na^+^/ K^+^ balance is also intimately related to renal control of acid-base balance. The K^+^ loss that occurs with many diuretics may lead to hypokalemia. For this reason, generally potassium-sparing diuretics are recommended^22^. In present study, hydroalcoholic extract of both *R. repens* and *R. pectinata* showed elevated levels of K^+^ in urine, which may increase risk of hypokalemia, and hence its potassium sparing capacity has to be investigated. Active phytoprinciples such as flavonoids, and terpenoids are known to be responsible for diuretic activity^23^–^25^. Isolation of these active principles and study of their exact mechanism of action needs to be investigated.

The ethanol extract of both *R. pectinata* and *R. repens* exhibited significant activity against all the tested bacteria and fungus. Acetone extract showed significant action against *Candida albicans* and only moderate action against other tested microorganisms. All the extracts of *R. pectinata* and *R. repens* showed moderate effects but chloroform and acetone extract of *R. repens* and ethanol extract of *R. pectinata* showed significant activity against *Trichophyton mentagrophytes*.
